# Prolactin as a Candidate Biomarker in Non-Small Cell Lung Cancer: Implications for Personalized Medicine and Post-Treatment Risk Stratification

**DOI:** 10.3390/jpm16070342

**Published:** 2026-06-24

**Authors:** Filip Gajewski, Grzegorz Kurec, Aleksandra Litkowska, Joanna Pec, Jakub Kleinrok, Weronika Pająk, Oliwia Burdan, Paweł Krawczyk, Agnieszka Korolczuk

**Affiliations:** 1 Department of Pneumonology, Oncology and Allergology, Medical University of Lublin, Doktora Kazimierza Jaczewskiego 8, 20-090 Lublin, Polanda_litkowska@interia.pl (A.L.); apec1410@gmail.com (J.P.);; 2 Chair and Department of Clinical Pathomorphology at Medical University of Lublin, Kazimierza Jaczewskiego 8b, 20-090 Lublin, Poland; 3 Laboratory of Immunology and Genetics, Medical University of Lublin, Doktora Kazimierza Jaczewskiego 8, 20-090 Lublin, Poland

**Keywords:** non-small cell lung cancer, prolactin, biomarker, personalized medicine, circulating biomarkers, risk stratification, prognosis, treatment monitoring, hyperprolactinemia, PRLR signaling

## Abstract

**Background/Objectives:** Non-small cell lung cancer (NSCLC) remains associated with high mortality, frequent late-stage diagnosis, biological heterogeneity, and recurrence after treatment. Although molecular and immunohistochemical biomarkers have transformed treatment selection, there remains a need for accessible, repeatable, and clinically practical circulating biomarkers that may support prognosis and post-treatment monitoring. This review discusses prolactin (PRL) as a candidate supplementary biomarker in NSCLC, with particular emphasis on its biological rationale, potential prognostic relevance, and possible role in personalized risk stratification after systemic therapy. **Methods:** This narrative review summarizes current evidence on established biomarkers in NSCLC, the physiology and regulation of PRL, PRL/PRLR signaling in cancer biology, mechanisms of PRL dysregulation in lung cancer, and available clinical observations concerning PRL alterations in NSCLC. Particular attention is given to the distinction between prognostic and predictive biomarkers, longitudinal monitoring, pituitary involvement, immune checkpoint inhibitor-related endocrine effects, and biological, pharmacological, and analytical confounders affecting PRL interpretation. **Results:** Current evidence suggests that PRL may be biologically relevant in NSCLC through its involvement in pathways related to cell proliferation, survival, angiogenesis, invasion, epithelial–mesenchymal transition, immune modulation, and possible therapy resistance. Clinical observations indicate that altered PRL levels may occur in advanced disease, pituitary involvement, systemic inflammation, stress, or during anticancer and supportive treatment. However, PRL lacks cancer specificity and is influenced by multiple confounders, including circadian rhythm, stress, endocrine disorders, macroprolactin, cachexia, medications, and assay variability. Available clinical data remain limited and are largely derived from small studies or case-based evidence. **Conclusions:** PRL should not currently be considered a standalone diagnostic, predictive, or treatment-selective biomarker in NSCLC. Its most realistic potential role is as a supplementary circulating marker within multimarker prognostic and monitoring models. Prospective validation with standardized sampling, assay procedures, and confounder adjustment is required before clinical implementation.

## 1. Introduction

### Global Burden and Clinical Significance of NSCLC

Lung cancer remains one of the most significant challenges in modern oncology, due to its prevalence, high mortality rate, and the rising number of cases among younger patients. Non-small cell lung cancer (NSCLC) is of particular significance, accounting for approximately 85% of lung cancer cases and primarily comprising adenocarcinoma, squamous cell carcinoma and large cell carcinoma [1,2,3].

Despite significant diagnostic and therapeutic advances, NSCLC continues to be associated with high mortality. This is due to several factors. A significant proportion of cases are diagnosed at a locally advanced or metastatic stage. Clinical symptoms in the early stages are often non-specific, and the disease is characterised by high biological heterogeneity. Furthermore, even after radical treatment, some patients experience recurrence [3].

In oncology, biomarkers are of particular importance because they enable a shift from treatment based solely on histopathological diagnosis to a management approach tailored to the biology of a specific tumour and the patient’s characteristics. Their use in personalised medicine stems not only from the possibility of selecting treatment methods, but also from more vigilant, objective monitoring following therapy [4,5].

The characteristics that an ideal clinical biomarker should possess are reliability, reproducibility, easy access, relatively low cost, the ability to be measured in routine practice, and ease of interpretation in the context of prognosis and therapeutic decisions. For this reason, there is growing interest in circulating biomarkers, which can be measured repeatedly and non-invasively [6].

Currently, many key NSCLC biomarkers rely on the analysis of tissue samples. Histopathological and molecular examination of the tumour remains essential, but has significant limitations. Biopsy material may be insufficient in terms of quantity or quality; a single biopsy does not always reflect the full heterogeneity of the tumour, and re-sampling tissue in the event of disease progression is often impossible or burdensome for the patient.

This clinical gap presents an opportunity for potential endocrine markers. The endocrine system influences proliferation, metabolism, inflammation, anti-tumour immunity, and the body’s stress response. This means that selected hormones may not only reflect the patient’s general condition but also participate in mechanisms promoting tumour progression. With regard to NSCLC, markers that can be measured in serum, which may change in response to treatment and which potentially correlate with clinical outcome following chemotherapy, are of particular interest.

Personalised medicine has significantly transformed the treatment of NSCLC, particularly through molecular and immunohistochemical testing. Nevertheless, there remains a need for markers that are inexpensive, reproducible, easy to measure, and useful for assessing prognosis and monitoring patients following treatment.

## 2. Scope of the Review

The aim of this review is to evaluate the potential role of prolactin as a supplementary circulating biomarker in patients with NSCLC, with particular emphasis on its prognostic significance following systemic treatment, including chemotherapy. We discuss the biological rationale for PRL involvement in NSCLC, summarize the available clinical evidence, and highlight the methodological challenges and future research directions required before PRL can be considered for routine clinical application. PRL is not currently recognised as a diagnostic marker or a factor determining treatment selection. Therefore, this review primarily analyses it as a potential adjunctive marker.

## 3. Personalized Medicine in NSCLC: Current Biomarker Landscape and Remaining Gaps

### 3.1. Molecular Biomarkers Currently Used in NSCLC

Molecular profiling has become a central component of personalized treatment in NSCLC, particularly in patients with advanced non-squamous tumors. The most clinically relevant actionable alterations include mutations in the EGFR gene, rearrangements involving ALK and ROS1 proto-oncogene 1, BRAF p.(Val600Glu) mutations, MET exon 14 skipping alterations, RET proto-oncogene fusions, NTRK fusions, KRAS p.(Gly12Cys) mutations, and selected human epidermal growth factor receptor 2 (HER2/ERBB2) alterations [7,8,9,10]. These biomarkers are primarily predictive, as their identification allows the selection of molecularly targeted therapies that may provide substantial clinical benefit compared with non-selective systemic treatment [11,12]. In addition to genomic alterations, PD-L expression remains an important immunohistochemical biomarker used to guide the selection of immune checkpoint inhibitor-based therapy, especially in tumors without actionable driver alterations, although its interpretation may be affected by tumour heterogeneity, assay variability, and the broader immune context of the tumour microenvironment (TME) [11,13].

Broad-panel next-generation sequencing (NGS), performed on tissue and increasingly supported by liquid biopsy when tissue is insufficient, is therefore recommended to identify actionable alterations efficiently and to avoid sequential single-gene testing [9,10]. However, despite the major therapeutic impact of these biomarkers, they do not fully address prognosis, longitudinal treatment monitoring, or risk assessment after systemic therapy.

Although tissue biopsy remains the gold standard for molecular profiling in NSCLC, alternative specimen types have also been explored. In particular, bronchoalveolar lavage fluid (BALF) has emerged as a potential source of tumor-derived material, including cell-free DNA, extracellular vesicles, proteins, and other biomolecules. Several studies have demonstrated the feasibility of detecting actionable genomic alterations, such as EGFR mutations, using BALF, with some reports suggesting higher sensitivity than plasma-based liquid biopsy in selected clinical settings. However, despite these promising findings, BALF-based molecular analyses have not yet been incorporated into routine biomarker testing algorithms because of limited standardization, heterogeneous methodologies, and insufficient prospective validation. Therefore, BALF should currently be regarded as an investigational complementary source of molecular information rather than an established alternative to tissue-based testing in NSCLC [14,15,16].

### 3.2. Prognostic Versus Predictive Biomarkers in NSCLC

In NSCLC, the distinction between prognostic and predictive biomarkers is essential for the correct interpretation of biomarker studies. A prognostic biomarker provides information about the likely clinical outcome of the disease, such as overall survival (OS), progression-free survival (PFS), recurrence risk, or disease aggressiveness, independently of a specific therapeutic intervention [12,17]. In contrast, a predictive biomarker indicates the likelihood of response or resistance to a particular treatment and is therefore directly linked to therapeutic decision-making [12,17,18].

This distinction is particularly important during the evaluation of prolactin as a potential biomarker in NSCLC. If elevated prolactin levels are associated with shorter survival or higher risk of progression, prolactin should be interpreted primarily as a prognostic biomarker [13]. However, to define prolactin as a predictive biomarker, studies would need to demonstrate that prolactin levels modify the probability of benefit from a specific treatment, such as chemotherapy, immunotherapy, or targeted therapy.

### 3.3. Established Blood-Based and Serum Biomarkers

Several blood-based biomarkers have been evaluated in NSCLC, mainly as supportive tools for prognosis and treatment monitoring rather than as independent diagnostic markers. The most frequently studied serum tumour markers include CEA, CYFRA 21-1, SCC antigen, and NSE [19,20]. CEA is more frequently associated with adenocarcinoma, CYFRA 21-1 and SCC antigen with squamous cell carcinoma, whereas NSE is primarily linked to neuroendocrine differentiation and small cell lung cancer, although it may occasionally be assessed in NSCLC [19,20].

Among these markers, CEA and CYFRA 21-1 have the strongest evidence for prognostic and monitoring utility. Elevated baseline levels have been associated with higher tumour burden and poorer outcomes, while decreases during systemic therapy may correlate with treatment response and radiological disease control [21,22]. However, their clinical value remains limited by insufficient sensitivity and specificity, histology-dependent performance, variable cut-off values, and possible elevation in non-malignant diseases or other cancers [19,20,21,22].

In parallel, systemic inflammatory and nutritional markers, including the NLR, CRP, albumin, CRP/albumin ratio, and LDH, have been explored as inexpensive prognostic indicators in NSCLC [23]. These parameters reflect host inflammation, immune status, nutritional decline, and disease burden, but they are highly non-specific and lack standardized thresholds. Therefore, although serum and inflammatory biomarkers are clinically attractive because they are inexpensive and repeatable, their limitations support the search for additional circulating markers, such as prolactin, that could complement existing prognostic models rather than replace established biomarkers.

### 3.4. Limitations of Current Biomarkers and Remaining Gaps in Personalized Monitoring

Despite major progress in molecularly guided treatment, current biomarkers in NSCLC do not fully address all clinical needs. Genomic alterations such as EGFR mutations, ALK and ROS1 rearrangements, BRAF, MET, RET, NTRK, KRAS, and HER2/ERBB2 alterations are highly valuable for treatment selection, but they mainly function as predictive biomarkers and are not always sufficient for continuous assessment of disease dynamics or post-treatment risk [8,9,10]. Similarly, PD-L1 expression supports immunotherapy selection, but its interpretation is limited by spatial and temporal heterogeneity, assay variability, and imperfect prediction of clinical benefit [11,19].

Classical serum biomarkers, including CEA, CYFRA 21-1, SCC antigen, and NSE, are inexpensive and repeatable, yet their sensitivity, specificity, and clinical interpretability remain limited [19,20,21,22]. Inflammatory and nutritional indices, such as NLR, CRP, albumin-based markers, and LDH, may provide additional prognostic information, but they are non-specific and strongly influenced by systemic conditions unrelated to cancer [23].

Therefore, an important gap remains in NSCLC personalized medicine: the need for accessible, low-cost, repeatable biomarkers that could complement molecular, immunohistochemical, and serum tumor markers in risk assessment and longitudinal monitoring. In this context, prolactin should not be viewed as a replacement for established biomarkers, but as a potential supplementary circulating marker that may contribute to broader multimarker prognostic models.

Given the biological complexity of prolactin regulation discussed in later sections, future research should investigate whether PRL can improve the performance of existing serum biomarker panels through multimarker predictive algorithms.

## 4. Physiology and Regulation of Prolactin

### 4.1. Prolactin Synthesis, Secretion and Neuroendocrine Regulation

Prolactin is a peptide hormone with a molecular weight of 23 kDa, consisting of 199 amino acids. In humans, prolactin is encoded by a single gene located on chromosome 6. It has a 3D structure composed of four anti-parallel α-helices, and thus exhibits strong structural homology not only with placental lactogen but also with growth hormone (GH) [23]. It is formed by the proteolytic cleavage of the signal peptide from the prohormone of prolactin, known as proprolactin [24].

The primary source of PRL is the pituitary gland. Lactophores located in the anterior lobe of the pituitary gland are responsible for the synthesis and release of prolactin [25]. PRL secretion is regulated by various factors. The pattern of PRL secretion is linked to the circadian rhythm. In humans, the highest levels are typically observed at night, between 2:00 and 4:00 a.m. During the day, PRL levels decrease [26]. The primary neuroendocrine factor regulating prolactin secretion is dopamine (DA). DA released by tuberoinfundibular dopamine (TIDA) neurons in the hypothalamus binds to D2 receptors on lactophores, thereby inhibiting dopamine synthesis and secretion [27,28].

In contrast, factors such as thyrotropin-releasing hormone (TRH), estrogen, and dopamine antagonists stimulate PRL secretion [24]. TRH has a stimulating effect on PRL secretion. It stimulates the lactotrophic cells of the pituitary gland [27]. Estrogens also play a role in stimulating PRL secretion. They directly stimulate lactophores and weaken the inhibitory effect of DA [27,29]. The secretion of PRL also increases in stressful situations. Stress is one of the physiological stimuli that increases its secretion. Its effects are associated, among other things, with a reduction in the activity of dopaminergic neurons through the action of endogenous opioids via the μ, κ, and δ opioid receptors [30].

Hyperprolactinemia can be physiological, pharmacological, or pathological in nature [26,31]. Physiological causes include pregnancy, lactation, stress, and nipple stimulation [31]. The most common pharmacological causes are antipsychotic medications, as well as certain antidepressants or opioids, which disrupt dopaminergic transmission [26,30]. The most common pathological cause is prolactinoma, but other pathologies are also mentioned, such as hypothyroidism or other tumors of the hypothalamic–pituitary region [31,32].

PRL is also synthesized outside the pituitary gland. Its synthesis is observed in peripheral tissues such as the endometrium, mammary gland, brain, and immune system cells. Extrapituitary prolactin exerts primarily local, autocrine, and paracrine effects. The regulation of its secretion does not depend on the hypothalamic–pituitary axis [33].

#### Physiological Functions of PRL

One of the main roles of prolactin is its mammogenic effect, that is, stimulating the growth and development of the mammary glands, as well as its lactogenic effect, which induces lactation, and its galactopoietic effect, which helps maintain milk production [34,35]. PRL influences milk production by inducing the enzyme that synthesizes milk components such as casein, lactose, and lipids [24].

PRL also has an effect on the hypothalamic–pituitary–gonadal axis, as it can, with pulsatility, inhibit the release of gonadotropin-releasing hormone (GnRH) by the hypothalamus [34].

Within the immune system, PRL may function similarly to a cytokine. It may play a role in both the innate and adaptive immune responses [36]. This hormone may influence the proliferation and survival of immune cells. Its potential role in autoimmunity is also emphasized, where it may disrupt the activity of regulatory T cells (Treg) and B-cell tolerance, lowering their activation threshold in a state of anergy [37].

PRL also influences metabolic processes in the body. Its effects depend, among other factors, on its concentration. Both a significant excess and a deficiency of prolactin can have a negative impact on metabolism, leading to excess body weight and disturbances in lipid and carbohydrate metabolism [38,39].

PRL also plays a role in regulating cell proliferation and differentiation through prolactin receptor (PRLR)-dependent signaling [40]. This is significant in carcinogenesis. Hormone-dependent cancers, such as breast, ovarian, endometrial, or prostate cancer, may be characterized by enhanced prolactin signaling [41].

An additional aspect that should be considered when evaluating prolactin as a potential biomarker is the existence of biologically distinct prolactin isoforms and proteolytic fragments. In addition to the full-length 23 kDa form, prolactin can undergo enzymatic cleavage to generate smaller peptides collectively referred to as vasoinhibins. These fragments exhibit biological activities that differ substantially from those of native prolactin, particularly with regard to vascular regulation. Vasoinhibins have been shown to exert anti-angiogenic, anti-vasopermeability, and pro-apoptotic effects on endothelial cells, partly through interactions with integrin α5β1 and the modulation of endothelial signaling pathways [42]. More recent evidence has highlighted the importance of the prolactin/vasoinhibin axis in cardiovascular diseases, emphasizing the distinct physiological and pathological roles of these peptide forms [43]. Given that angiogenesis is a fundamental process in NSCLC progression and metastatic dissemination, the relative balance between full-length prolactin and vasoinhibins may have important implications for tumour biology. Therefore, future studies investigating prolactin as a biomarker in NSCLC should consider not only total circulating prolactin concentrations but also the potential contribution of specific prolactin isoforms and their differential biological activities.

### 4.2. PRLR Isoforms and Intracellular Signaling Pathways

PRLR is linked to the non-receptor tyrosine kinase Janus 2 (JAK2) and is a member of the class I cytokine receptor family. It exists in several isoforms that differ in their functional and structural properties [44]. Three isoforms of the PRLR have been described in humans: long, short, and intermediate. These isoforms arise from alternative splicing of the primary transcript. The differences between the isoforms concern variations in the length of the amino acid chain in the intracellular region. The transmembrane domains and extracellular regions remain the same [45]. This affects their varying ability to activate signaling pathways. PRL signaling seems to be significantly influenced by the long PRLR isoform. Many kinases, including JAK2, mitogen-activated protein kinase (MAPK), and phosphoinositide 3-kinase (PI3K), are activated when PRL binds to this receptor. Cell survival, differentiation, and proliferation are all aided by their activation. A frameshift mutation produces the intermediate isoform, which likewise activates the Janus kinase/signal transducer and activator of transcription (JAK/STAT) pathway but cannot promote cell division [44]. PRL, by binding to PRLR, leads to the activation of several key signaling pathways associated with tumorigenesis, such as JAK/STAT, mitogen-activated protein kinase/extracellular signal-regulated kinase (MAPK/ERK), and phosphoinositide 3-kinase/protein kinase B (PI3K/AKT) [41].

The JAK/STAT pathway is considered the canonical signaling pathway activated upon PRLR activation. It is particularly associated with its long isoform. Following the binding of PRL to PRLR, JAK2 is activated, STAT proteins are phosphorylated, and subsequently translocated to the cell nucleus, where they regulate the expression of target genes. Activation of this pathway promotes cell differentiation, proliferation, and survival. Particular importance is attributed to the STAT5A and STAT5B proteins [41,44].

The RAS/MAPK/ERK pathway is also associated with prolactin signaling. It is linked to neoplastic transformation and cell proliferation [41,46]. Abramicheva et al. indicate that the MAPK/ERK pathway can be activated by both the long and short isoforms of PRLR [44]. More recent data suggest that the intermediate isoform of PRLR may contribute to breast cancer oncogenesis by activating the RAS/MAPK/ERK pathway [47].

Prolactin signaling is also significantly influenced by the PI3K/AKT pathway [45]. Cell growth, proliferation, and survival can all be impacted by PI3K/AKT activation [44]. This pathway may be one of the primary pathways of PRLR signaling in prolactin-dependent tumors and other malignancies [45].

**Figure 1:**
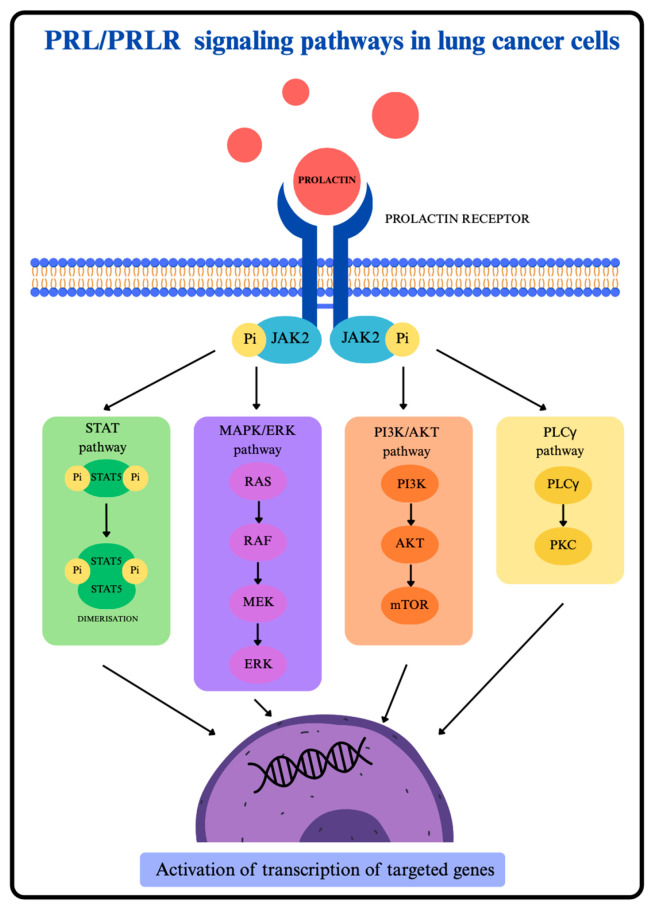
PRL/PRLR signaling pathways implicated in NSCLC biology. Binding of prolactin (PRL) to the prolactin receptor (PRLR) activates JAK2 and downstream signaling cascades, including the JAK/STAT, MAPK/ERK, PI3K/AKT/mTOR, and PLCγ pathways. These pathways regulate transcriptional programs involved in cell proliferation, survival, differentiation, and tumour progression.

PRLR expression exhibits tissue specificity, which enables tissue-specific responses to PRL that also depend on the organism’s physiological state. The presence of the receptor has been described in many tissues and organs involved in the regulation of water and electrolyte balance, growth and development, endocrine and metabolic functions, brain activity and behavior, reproduction, and the immune response [48,49]. Disorders of the PRL/PRLR axis have been described in tumors, particularly hormone-dependent ones. It is worth noting, however, that research indicates this pathway may also play a role in promoting the development of other types of cancer, including liver, colorectal, and pancreatic cancer [41]. Not only the expression levels of individual PRLR isoforms, but also the ratio between them and the dominance of a specific isoform may have biological significance. In Abramicheva’s study, the researchers emphasize that the long PRLR isoform may promote cell proliferation and survival, whereas some short isoforms may exhibit antiproliferative and proapoptotic effects [23]. In the study by Standing et al., the researchers suggest that the intermediate PRLR isoform may be of particular importance, as it has been associated with increased proliferation, viability, and transformation of breast cancer cells (Figure 1) [41].

#### Prolactin in Tumor-Related Processes

As it was mentioned before, PRL is considered a pleiotropic hormone whose effects surpass classic endocrine functions. In the cancer context, PRL regulates multiple key biological processes such as cell proliferation, cell survival, angiogenesis, and migration and modulation of the TME.

PRL has been shown to have an impact on tumor proliferation by the activation of the PRL-PRLR pathway, which leads to phosphorylation of JAK2 and further activation of STAT5 transcriptional factors, which regulate gene expression and cell survival. At the same time, the activated PI3K/AKT pathway promotes cell survival by inhibiting proapoptotic proteins and activating mTOR, favouring cell growth. It has also been reported that PRL may modulate tumor cell response to oxidative stress and DNA impairment, enhancing their capability of survival during treatment. Therefore, it suggests that PRL may have an impact on treatment resistance development, especially chemotherapy and targeted therapies [41,50,51,52].

What is more, PRL may influence angiogenesis. A full-length PRL plays a proangiogenic role via factors such as VEGF, whereas proteolytic fragments—vasoinhibins—may have an antiangiogenic role. This dual role shows that the PRL effect on tumor angiogenesis may depend on local TME, activity of proteolytic enzymes, and the balance between different isoforms of the hormone [53,54,55,56].

Multiple studies report that PRL may influence the ability of tumor cells to migrate and invade by regulating cell adhesion and extracellular matrix degradation. Activation of the MAPK/ERK and PI3K/AKT pathways leads to increased expression of metalloproteinases (MMP-2, MMP-9), which play a key role in tissue invasion [57,58,59].

Furthermore, evidence suggests that PRL may participate in the regulation of epithelial–mesenchymal transition (EMT). These mechanisms include the modulation of the expression of adhesion proteins and transcription factors associated with EMT. Despite the limited research for NSCLC, observations from other tumors support the hypothesis of a potential role for PRL in promoting a more aggressive phenotype [50,60].

PRL also plays a significant role in shaping the TME through its immunomodulatory abilities. It can influence the function of T cells, NK cells, macrophages, and dendritic cells, modulating both the antitumor response and immunosuppressive mechanisms. Particularly, PRL may promote the differentiation of macrophages toward the M2 phenotype, which favors tumor progression, angiogenesis, and suppression of the immune response. Additionally, PRL may influence the production of cytokines and chemokines, thereby modifying communication between tumor cells and components of the microenvironment [61,62,63].

NSCLC may influence the hypothalamic–pituitary axis through paraneoplastic mechanisms and chronic activation of the stress axis. Disruptions in the dopaminergic control of PRL secretion can lead to its overproduction in the course of cancer. Moreover, proinflammatory cytokines may stimulate PRL secretion both on the pituitary level and in the peripheral endocrine tissues, highlighting the connection between immune and endocrine systems [62,63,64]. Secretion of proinflammatory cytokines, such as IL-6, affects neuroendocrine pathways and impairs hormone regulation, leading to high PRL levels. PRL itself presents immunomodulatory effects and may modulate signal pathways such as JAK/STAT, MAPK/ERK or PI3K/AKT, suggesting its potential role not only as a biomarker but also as an active participant of tumor processes [64,65].

In the context of paraneoplastic syndromes, it should be highlighted that despite ectopic hormone production in lung cancer concerning mostly ACTH or ADH, the systemic effects of cytokines and growth factors produced by the tumor can lead to impairments in the hypothalamic–pituitary axis, thereby affecting prolactin secretion [65,66]. What is more, chronic activation of the stress axis (HPA) in patients with advanced NSCLC may lead to hormonal dysregulation. Disruptions of this axis, also observed during immunotherapy, affect the balance between cortisol and the dopaminergic system, which in turn may modulate PRL secretion [66,67].

### 4.3. Mechanisms of Prolactin Dysregulation in Lung Cancer

PRL dysregulation in patients with NSCLC is an effect of multiple endocrine, inflammatory and iatrogenic mechanisms. In contrast to other hormone-dependent tumors, in NSCLC, changes in the PRL levels mirror a complex response to the disease rather than one pathological process [64,68].

#### 4.3.1. Therapy-Related Effects on Prolactin Levels

Lung cancer treatment is an important element influencing the PRL levels. The effect is complex and dependent on the type of treatment [69,70]. Chemotherapy may indirectly influence PRL secretion through inflammatory response induction and metabolic impairments [71]. Even though the direct impact of chemotherapy on the pituitary gland is not conclusive, the systemic nature of the treatment contributes to hormonal homeostasis disorders [70]. Other treatment methods that may lead to high PRL levels include antipsychotic treatment, which is used in oncological patients. The usage of antipsychotic drugs is reported to raise the PRL levels [72,73]. Opioids, commonly used in the treatment of cancer pain, may contribute to high PRL levels by inhibiting the activity of dopaminergic neurons [74]. Glucocorticoids affect the HPA axis, and their impact on PRL levels remains variable and context-dependent [75]. Recently, attention has been drawn to the potential impact of immunotherapy on hormonal balance. Immune checkpoint inhibitors (ICIs), such as anti-PD-1 and anti-PD-L1 antibodies used in NSCLC, may induce endocrine disorders. Reports have emerged suggesting the possibility of high PRL levels during treatment with nivolumab. The mechanism behind it is not fully understood, but it is believed to result from an interaction between the immune and endocrine systems, in which prolactin acts as a cytokine modulating lymphocyte activity [76,77,78,79].

#### 4.3.2. Inflammation, Stress, and Systemic Illness as Mediators

**Figure 2:**
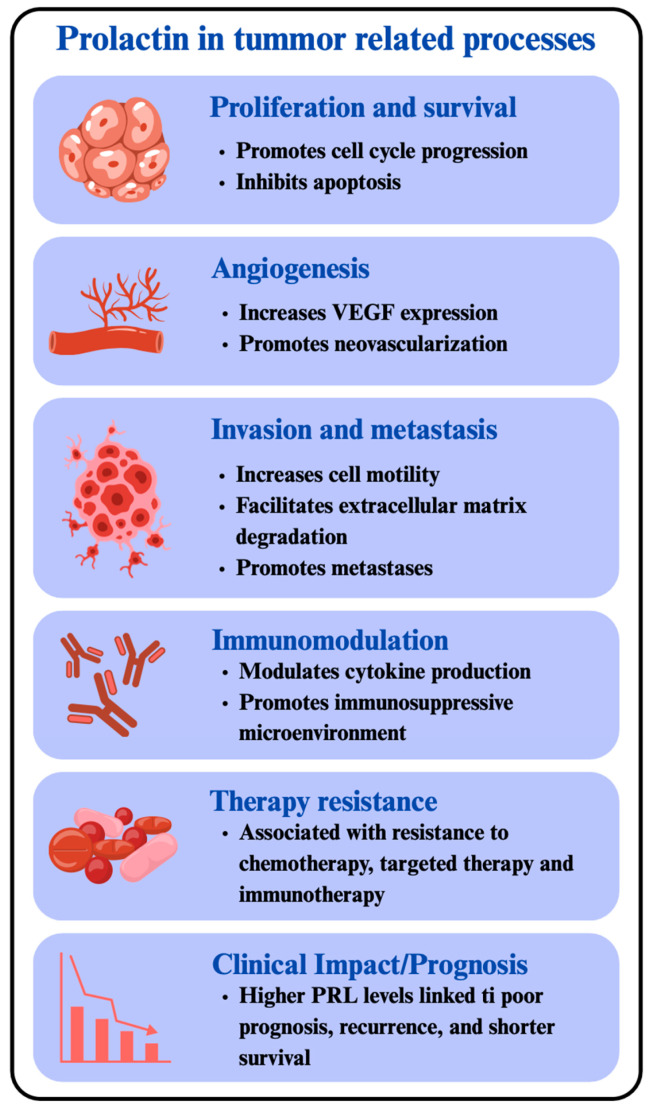
Proposed roles of prolactin in tumour-related processes relevant to NSCLC. Experimental evidence suggests that prolactin signaling contributes to tumour cell proliferation and survival, angiogenesis, invasion and metastatic dissemination, modulation of the tumour microenvironment, therapy resistance, and adverse clinical outcomes. The figure summarizes the potential biological and clinical implications of PRL dysregulation in NSCLC.

An important role of PRL regulation in NSCLC is played by chronic inflammation, immune response, and the general condition of the patient. NSCLC presents a strong inflammatory component where proinflammatory cytokines, including interleukins and TNF-α, may influence PRL secretion both directly and through modulation of the hypothalamic–pituitary axis [80]. Therefore, a stress response may play a crucial role as a PRL-secretion mediator. Activation of the HPA axis in response to chronic cancer-related stress leads to changes in cortisol secretion and neurotransmitter imbalances, including dopamine, which secondarily influence prolactin secretion. What is more, malnutrition and cancer cachexia further enhance endocrine disruptions. Metabolic disturbances affect the functioning of endocrine axes, including PRL regulation (Figure 2) [81,82,83].

### 4.4. Integrative Multimarker Predictive Modeling Incorporating Prolactin

Given that prolactin is influenced by systemic inflammation, stress, pharmacotherapy, and comorbid conditions, isolated PRL elevations may introduce substantial biological variability and reduce its interpretative specificity in NSCLC [23,30,80]. For this reason, its clinical utility is likely to be most robust when assessed in combination with other biomarkers rather than in isolation.

A practical approach is the integration of PRL into multimarker predictive models together with established tumor-associated biomarkers such as CEA, CYFRA 21-1, NSE, and SCC antigen [19,20,21,22]. In this framework, biomarkers contribute weighted information within a unified predictive score for diagnostic, prognostic, or monitoring purposes.

From a methodological perspective, multivariable regression models (e.g., logistic regression and Cox proportional hazards models) enable estimation of independent biomarker contributions while accounting for clinical covariates [21,23]. In parallel, machine learning approaches, including penalized regression (e.g., LASSO), random forests, and gradient boosting methods, may improve model performance by capturing nonlinear relationships and mitigating the impact of biologically variable markers.

The rationale for such integration is the complementary nature of the included biomarkers: classical tumor markers primarily reflect tumor burden and histological subtype, whereas PRL may provide additional information on systemic endocrine–immune and tumor–host interactions. Their combined assessment may therefore improve discrimination between tumor-associated biological changes and nonspecific fluctuations in PRL levels.

These multimarker strategies require rigorous validation and calibration across independent cohorts but are increasingly applied in oncology to enhance predictive accuracy beyond single-marker approaches [11,21,23].

## 5. Clinical Evidence on Prolactin in NSCLC

### 5.1. Clinical Observations and Methodological Challenges in Prolactin Assessment

Current clinical data suggest that prolactin serves a functional role across various malignancies, including lung cancer, rather than acting as a mere physiological bystander. Consequently, its presence in patients with non-small cell lung cancer should not be dismissed as an incidental finding, but rather viewed as a component of a sophisticated oncogenic mechanism. When evaluating the frequency of hyperprolactinemia, we must account for three distinct sources of the hormone identified in the recent literature: classic endocrine secretion from the pituitary gland, autocrine and paracrine production by the neoplastic epithelium itself, and stromal-sourced secretion. This latter factor is particularly noteworthy, as cells within the tumor microenvironment have been shown to synthesize PRL, thereby driving tumor progression. The variability in reported hyperprolactinemia rates likely stems from a fundamental methodological gap: standard assays measure systemic, circulating PRL while overlooking localized production within the tumor. Such a limitation may lead to a significant underestimation of the hormone’s biological impact in a substantial subset of patients [60].

Estimates of hyperprolactinemia frequency in lung cancer remain scattered throughout the literature. However, cohort studies consistently reveal significant elevations when compared to healthy populations. While systemic dysregulation is common, direct structural damage to the endocrine axis must also be considered in advanced cases. Autopsy data indicate that pituitary metastases occur in 1% to 4% of patients with terminal malignancies, a factor that may further complicate the interpretation of prolactinemia in the final stages of the disease [84]. These lesions account for approximately 1% of all pituitary tumors and roughly 0.4% of all intracranial metastatic growth. Interestingly, while autopsy findings suggest a higher prevalence, the clinical frequency diagnosed during a patient’s lifetime remains near 1% [85]. Lung and breast cancers combined account for approximately 50% of all pituitary metastases, with the highest incidence observed in patients aged 60 to 70 years. Recent reports highlight an increasing frequency of anterior hypopituitarism among those with metastatic involvement of the gland. In one clinical case involving a patient with metastatic adenocarcinoma, mild hyperprolactinemia was documented at 110 µIU/mL, significantly exceeding the reference range of 3.5–26 µIU/mL. Following the surgical resection of the tumor mass, prolactin levels plummeted to 20 µIU/mL. This rapid normalization underscores that the hyperprolactinemia was a direct consequence of mechanical compression of the pituitary gland or stalk by the tumor [84].

Clinical symptoms suggesting pituitary axis dysfunction, including those related to PRL, manifest in only about 20% of patients. This suggests that for the vast majority, hyperprolactinemia remains an undiagnosed, subclinical condition. While lung cancer stands as the leading cause of pituitary metastases, the actual prevalence of hyperprolactinemia in NSCLC patients without central nervous system involvement is poorly documented. This gap in the literature underscores a pressing need for broader screening within this population.

Another barrier lies in the phenomenon of macroprolactinemia—the presence of high-molecular-weight hormone isoforms that possess minimal biological activity yet remain detectable by standard immunoassays. As Biagetti et al. (2022) [86] point out, the lack of a universal requirement for polyethylene glycol (PEG) precipitation to eliminate macroprolactin is a primary driver of misdiagnosis and inconsistency in reported hyperprolactinemia rates. Within the oncological literature, it is rare to find clarification on whether results were corrected for the macroprolactin fraction. This oversight suggests that the true prevalence of biologically active hyperprolactinemia may be systematically overestimated across many patient cohorts [86].

The lack of a standardized cutoff for “oncological hyperprolactinemia”, particularly in patients dealing with cancer cachexia or chronic stress, creates a diagnostic gray area. A result of 30 ng/mL might be flagged as pathological in one cohort but dismissed as a reactive, normal finding in another. The pharmacological profile of concurrent medications is another critical driver of these reporting discrepancies. Recent analyses indicate that using prolactin-sparing agents, such as aripiprazole, results in significantly lower serum PRL levels compared to standard regimens involving potent dopamine antagonists. Such therapeutic variables must be carefully accounted for when comparing data across different patient cohorts [87].

The discrepancies in reported hyperprolactinemia rates among NSCLC patients stem largely from a lack of standardized research methodology. A primary challenge is the absence of uniform cut-off points. Ken-Dror et al. (2024) [88] demonstrated that even when studies focused on “low prolactin,” their definitions varied wildly: for men, “low” was defined as <3.6 ng/mL in one study, yet reached <7.2 ng/mL in another. Among women, this spread was even more pronounced, ranging from <4.5 ng/mL to <8 ng/mL. Laboratory techniques present a further barrier to consistency. Although all studies analyzed by Ken-Dror et al. (2024) [88] utilized chemiluminescence immunoassay technology, significant inconsistencies remained. Even with modern immunoassays, variations in equipment and reagents, such as those between Roche platforms and other manufacturers, mean that identical serum concentrations can lead to divergent clinical interpretations. Finally, the systemic failure to control for confounding variables remains a critical flaw in current data. Many studies overlook the impact of hospitalization-related stress, diurnal secretion fluctuations, and the influence of supportive pharmacotherapy. Without accounting for these external drivers, the true prevalence of tumor-associated hyperprolactinemia remains difficult to isolate [88].

The biological complexity of this axis is further deepened by the promiscuity of the prolactin receptor (PRLR), which can be activated by ligands other than PRL itself, such as growth hormone (GH) and placental lactogen. This cross-reactivity introduces a significant diagnostic blind spot: a patient may present with perfectly normal serum prolactin levels, yet their pulmonary PRLRs could be “bombarded” by GH. This alternative signaling pathway can trigger the same deleterious oncogenic effects as hyperprolactinemia, effectively masking the true intensity of the receptor’s activation in the tumor microenvironment (Table 1) [60].

#### Pre-Analytical Standardization Framework for Longitudinal Prolactin Assessment

To improve comparability of longitudinal prolactin measurements, a minimal standardized protocol is required to reduce pre-analytical variability and ensure clinically meaningful interpretation of temporal trends.

1. Pre-sampling conditions
  Patients should avoid intense physical activity and unnecessary stressors for 24 h prior to sampling. Blood collection should preferably be performed in a stable outpatient setting rather than during acute hospitalization whenever possible.
2. Medication and confounder control
  All concomitant therapies influencing prolactin secretion (e.g., antipsychotics, antidepressants, antiemetics, opioids) should be systematically documented at each time point, including recent dose changes.
3. Sampling procedure
  Morning fasting blood samples are recommended. A short rest period (15–30 min) prior to venipuncture should be ensured. For repeated measurements, the use of an indwelling venous catheter is preferred to minimize procedure-related variability.
4. Analytical consistency
  The same immunoassay platform should be used throughout follow-up when possible. Reporting should include whether macroprolactin screening (e.g., PEG precipitation) was performed.
5. Longitudinal design and interpretation
  Assessment should rely on predefined serial time points rather than isolated measurements. Interpretation should prioritize intra-individual trajectories over fixed population cut-offs, integrating clinical context and treatment status.

### 5.2. Prolactin Alterations in Advanced NSCLC and Pituitary Involvement

The dynamics of prolactin concentrations in lung cancer patients are intricately linked to both the disease stage and the specific therapeutic interventions employed. Clinical case analyses demonstrate that these hormonal parameters can fluctuate rapidly, positioning them as a sensitive indicator of the patient’s current clinical status. Evidence from clinical studies suggests a higher prevalence of elevated PRL levels in patients with advanced-stage NSCLC. In these settings, the hormone may function as more than a passive marker of tumor burden. It appears to act as an active promoter of dissemination. This association is driven by the direct involvement of prolactin in the molecular and cellular processes underlying metastasis [41].

During the diagnostic phase, particularly in metastatic disease, the hormonal profile can become profoundly disrupted. A striking example involves a patient with a pulmonary neuroendocrine tumor (NET) measuring 75 × 87 × 106 mm, whose pre-intervention prolactin levels reached “tumoral” ranges, oscillating between 330 and 832 ng/mL against a reference norm of 25 ng/mL. While such extreme elevations typically point toward a macroprolactinoma, the underlying cause here was an ectopic stimulatory mechanism. The lung tumor secreted massive quantities of GHRH (38,088 ng/L), triggering secondary lactotroph hyperplasia and enlarging the pituitary gland to 22 × 30 × 34 mm. This case demonstrates that in advanced disease, high prolactin can be a direct byproduct of the metabolic activity of the primary thoracic mass [90].

However, while the role of prolactin in late-stage malignancy is increasingly well-documented, the impact of early-stage, small nodules on the hormonal profile remains poorly understood and warrants prioritized clinical investigation. The most dramatic shifts in prolactin levels are observed following radical surgical intervention. Clinical case reports indicate that as early as five days post-resection, PRL levels can plummet from tumoral peaks to as low as 1.9 ng/mL. In the long term, as demonstrated in follow-up observations approximately nine months post-surgery, prolactin concentrations tend to stabilize within the physiological range (around 3.6 ng/mL), while the previously hyperplastic pituitary volume can regress by as much as 70%. The clinical validation of this systemic normalization is often evidenced by the return of spontaneous menstrual cycles in female patients. Such a radical transformation of endocrine parameters following lung surgery provides compelling evidence that the hyperprolactinemia was entirely tumor-dependent, positioning it as a dynamic marker of therapeutic efficacy [90].

The use of potent analgesics, specifically opioids, represents another significant variable in the hormonal landscape of oncological patients. A 2021 meta-analysis by Diasso et al. confirms that prolonged opioid administration profoundly disrupts the prolactin axis. This pharmacological interference adds a layer of complexity to blood test interpretations, as the resulting shifts in serum PRL levels may reflect drug-induced alterations rather than tumor dynamics alone [92]. In the context of tumor staging according to the 8th edition of the TNM (where T stands for primary tumor, N for regional lymph nodes, and M for distant metastasis) classification, the occurrence of NSCLC metastasis to the pituitary gland (M1 category) is of particular clinical significance. The presence of such a neoplastic mass profoundly alters the hormonal profile, with a rapid surge in prolactin levels—often exceeding 200 ng/mL—serving as a critical red flag. This phenomenon is driven by the “stalk effect,” where the tumor compresses the pituitary stalk and obstructs the dopaminergic flow that normally inhibits PRL secretion, compounded by the direct invasion of aggressive adenocarcinoma cells into the surrounding tissues. Clinically, such extreme hyperprolactinemia acts as a clear indicator of heavy systemic tumor burden and rapid disease progression. Unfortunately, these findings are synonymous with a poor prognosis. In such cases, the median survival rarely extends beyond a single year [93].

It is noteworthy that hyperprolactinemia in advanced NSCLC (stage IVB) is not always a baseline finding. It often emerges as a herald of disease progression during active therapy. This is vividly illustrated by a case involving a patient with an ALK gene fusion who, after maintaining a year of stability on crizotinib, experienced a sudden onset of neurological symptoms accompanied by a surge in prolactin to 113.35 ng/mL. Such a spike in a previously stable patient serves as a definitive clinical alarm, suggesting the emergence of new metastases—specifically to the pituitary in this instance, and a resultant increase in systemic tumor burden. In this case, the hyperprolactinemia occurred in tandem with the development of the secondary resistance mutation in the ALK gene (p.(Ile1171Ser). This suggests that hormonal shifts may indirectly mirror the genetic evolution of the tumor as it escapes the control of targeted therapies. The sensitivity of this marker is further evidenced by the immediate normalization of prolactin levels following the surgical resection of the metastatic lesion [89].

In clinical practice, the profile of a patient with pituitary metastasis typically centers around the age of 50, with a statistical mean of 49.5 years. While biological sex might not initially seem like a decisive factor, the data reveal a clear male predominance, with men accounting for 64% of cases. Furthermore, lung cancer is the undisputed “primary culprit,” responsible for nearly half of all metastases in this location—a figure that reaches exactly 50% in the male population. The most immediate “red flag” for a clinician is the combination of two critical factors: the patient’s oncological history and the rapid expansion of the intracranial mass. While a standard pituitary adenoma is benign and grows slowly over the years, a metastasis can increase in volume by 20% in just a few months, with some cases progressing in as little as 37 days. On MRI, these lesions are typically substantial, averaging 2.7 cm and often occupying the entire sella turcica. When a patient with a prior history of malignancy presents with a pituitary lesion exhibiting such explosive growth, the diagnostic certainty that it is a metastasis reaches 97.9%. Unfortunately, this aggressive biological behavior translates into a very challenging prognosis. From the moment of diagnosis, the median survival typically spans only six months [94].

### 5.3. Prolactin in Patients Treated with Immune Checkpoint Inhibitors

The etiology of pituitary dysfunction varies significantly depending on the histological subtype, disease dynamics, and the specific therapeutic strategy employed. While aggressive metastatic involvement is characterized by mechanical invasion and high prolactin levels, recent data reveal a different pattern in patients responding well to immunotherapy. In both adenocarcinoma and squamous cell carcinoma, treatment with PD-1 inhibitors, such as pembrolizumab or sintilimab, can trigger immune-related hypophysitis, leading to isolated ACTH deficiency. Interestingly, these endocrine complications often manifest while the primary tumor remains stable or in partial remission (PR), suggesting that a robust anti-tumor immune response may “erroneously” target pituitary tissue. A critical diagnostic distinction lies in the hormonal profile: unlike metastatic infiltration, where prolactin surges drastically, patients with immunotherapy-induced complications typically maintain normal prolactin levels. This stability is a vital clinical clue. The absence of a PRL spike in a patient presenting with symptoms of adrenal insufficiency—such as fatigue, nausea, or hyponatremia—allows clinicians to differentiate an autoimmune reaction from physical neoplastic infiltration. Furthermore, in these immunotherapeutic contexts, MRI scans of the pituitary often appear entirely unremarkable, showing no enlargement or stalk thickening despite severe hormonal deficiency. This reinforces the notion that the histological subtype and disease stage dictate whether pituitary damage is the result of mechanical destruction by a tumor or metabolic disruption caused by inflammation [91].

### 5.4. Prolactin in Immune-Related Pituitary Dysfunction

In patients with NSCLC treated with PD-1 inhibitors like pembrolizumab or sintilimab, stable prolactin levels serve as a pivotal tool for differential diagnosis. This stability allows clinicians to distinguish between immune-related complications, such as isolated ACTH deficiency (IAD), and pituitary metastases. While PRL typically remains within the normal range in the former, metastatic involvement usually triggers a rapid hormonal surge. Notably, these endocrine disturbances often coincide with a robust oncological response, including partial remission, which suggests that intense immune activation may inadvertently target the pituitary gland. Post-treatment monitoring requires vigilance for up to 17 months after the initial dose or for at least six months following the complete cessation of immunotherapy, as autoimmune mechanisms can manifest with significant latency.

Despite these promising clinical observations, the utility of prolactin in post-treatment monitoring for NSCLC remains constrained by a lack of large-scale prospective trials. Much of the current evidence is drawn from case reports or small cohorts, which preclude definitive statistical conclusions regarding the sensitivity of PRL when measured against established markers like CEA or CYFRA 21-1.

### 5.5. Prolactin, Tumour Progression, Metastatic Potential and Therapy Resistance

The role of prolactin in the progression of neoplastic diseases extends far beyond the stimulation of primary tumor growth, encompassing key processes that determine progression-free survival and the dynamics of metastatic dissemination. Prolactin contributes to the shortening of PFS by inducing resistance to DNA-damaging chemotherapeutic agents, such as doxorubicin and cisplatin. This mechanism relies on the interaction of the PRL-PRLR-JAK2-STAT5 signaling pathway with heat shock proteins (HSP90) and the DNA damage response system (ATM), which collectively promote cancer cell survival despite active treatment.

High serum PRL levels and the overexpression of its receptor directly correlate with shorter times to disease recurrence. Furthermore, the autocrine secretion of prolactin by tumor cells shifts their phenotype toward a more mesenchymal state, enhancing invasive capacity, facilitating migration, and promoting colony formation, which leads to the earlier emergence of new foci following radical treatment. The time to progression is further modified by external factors within the tumor microenvironment. For instance, the rigidity of the extracellular matrix—such as dense collagen—shifts PRL signaling from the JAK2/STAT5 pathway to the more aggressive MAPK/ERK route, intensifying invasiveness and accelerating clinical progression. Prolactin also activates RAS/RAF/MAPK/ERK signaling cascades, which are directly linked to invasiveness and the ability to form distant metastases. In cases of bone metastasis, PRL stimulates the secretion of factors like Sonic Hedgehog (SHH), triggering a “vicious cycle” of bone destruction and growth factor release that drastically hastens disease advancement in these areas. The metastatic potential is primarily driven by the long isoform of the PRLR. Research has shown that reducing the activity of this specific form inhibits the formation of lung and liver metastases, suggesting that the long PRLR isoform is a primary driver of oncological progression. In summary, prolactin shortens both progression-free and disease-free survival not only by fueling cell division but also by actively supporting invasiveness, facilitating the creation of metastatic niches, and building resistance to standard oncological therapies [60].

### 5.6. Limitations in the Prognostic Interpretation of Prolactin

The interpretation of prolactin’s role as a prognostic indicator in oncology demands a high degree of critical scrutiny, as elevated levels are not always a direct consequence of the tumor’s autonomous secretory activity. Mistaking oncological signals for an infection leads to critical delays in initiating appropriate therapy. Furthermore, aggressive disease progression accompanied by a sudden surge in prolactin can occur in young, non-smoking individuals harboring EGFR mutations. This supports the thesis that the prognostic value of prolactin is inextricably linked to the patient’s genetic profile and interactions within the tumor microenvironment. A prime example is the stiffness of the extracellular matrix, which can “switch” PRL signaling from differentiation pathways to pro-metastatic routes. A final limitation to reliable clinical inference is the inability of currently available diagnostic assays to distinguish between PRLR isoforms, despite evidence that the long PRLR isoform is specifically responsible for enhanced metastatic potential, tumor progression, and therapy resistance [95,96]. A major analytical challenge stems from the limitations of currently available immunoenzymatic and immunohistochemical assays. Most commercially available antibody-based methods are designed to detect conserved receptor domains shared by multiple PRLR isoforms and therefore measure total PRLR expression rather than the abundance of individual receptor variants. Because the long and short PRLR isoforms differ primarily in their intracellular domains and downstream signaling capacities, conventional assays cannot determine which isoform predominates within a given tumor. Consequently, patients with markedly different risks of recurrence and metastatic dissemination may be classified similarly based on overall PRLR positivity. This lack of isoform specificity reduces the prognostic value of PRLR assessment and may obscure clinically relevant differences in disease behavior.

### 5.7. Hormonal Parameters in Treatment Response Monitoring

The relationship between prolactin levels and therapeutic response is not uniform, as it depends heavily on the specific mechanism of action of the treatment and the genetic profile of the tumor (Table 2).

**Table 2:** Morphological and biological tumor characteristics vs. hormonal profiles in NSCLC [90,93]. Abbreviations: ACTH—adrenocorticotropic hormone; IHC—immunohistochemistry; Ki-67—proliferation marker; M1—distant metastasis; PRL—prolactin.

Tumor Characteristic	Description and Clinical Significance	Impact on Hormone Levels (PRL/ACTH)
Location and size	Strong correlation with high tumor burden and M1 stage. Tumor mass occupying the sella turcica physically affects pituitary structures.	Induces the so-called stalk effect; mechanical compression blocks dopamine delivery, resulting in a rapid surge of prolactin.
Grading and aggressiveness	High histological grade and significant proliferative activity (Ki-67 index at 5–10% of cells).	Promotes aggressive and rapid infiltration of intracranial structures, leading to sudden manifestation of hormonal symptoms.
Receptor and marker expression	Expression of neuroendocrine markers in metastatic cells confirmed by immunohistochemical (IHC) studies.	Suggests that hormonal disturbances may result from the tumor biology itself and its ability to secrete biologically active substances, rather than solely from mechanical compression.

Data analysis demonstrates that hormonal parameters may serve as a potential tool for monitoring NSCLC treatment outcomes. Elevated levels of prolactin and an excess of activin A function as early warning signals, heralding potential resistance to both immunotherapy (specifically nivolumab and atezolizumab) and platinum-based chemotherapy. Gender is a critical variable in this monitoring process. In female patients, superior immunotherapy outcomes may correlate with higher leptin levels, even though estrogens tend to impair PD-L1 expression. Furthermore, a specific clinical paradox is observed during the administration of PD-1 inhibitors: a decline in ACTH levels coupled with normal prolactin concentrations often confirms therapeutic efficacy and tumor destruction, despite the induction of hormonal complications. Additionally, because PRL, FSH, and LH promote tumor invasiveness by blocking the HO-1 molecule, achieving the stabilization of these hormonal levels is essential for securing sustained clinical improvement [91] (Table 3).

## 6. Potential Clinical Utility of Prolactin in Personalized Medicine

Although molecular profiling has substantially improved treatment selection in NSCLC, current biomarkers do not fully address the need for accessible, repeatable, and clinically practical tools for prognostic assessment and longitudinal monitoring [18,97]. In this context, PRL should not be interpreted as an independent diagnostic or therapeutic decision-making marker but rather as a potential supplementary circulating biomarker that may add biological and clinical information to existing models [18,50]. Its possible value results from the combination of several features: low-cost serum measurement, biological links with cell proliferation and survival pathways in NSCLC, and preliminary clinical observations suggesting associations between PRL alterations and disease course during systemic therapy [50,76]. Therefore, the potential clinical utility of PRL in personalized medicine should be considered mainly in three areas: risk stratification, longitudinal monitoring, and identification of patient subgroups in whom hormonal or inflammatory dysregulation may reflect more aggressive disease biology [18,21,50,76].

### 6.1. Risk Stratification

PRL may be considered a potential supplementary marker for risk stratification in NSCLC, particularly when interpreted together with established clinical and laboratory variables. Its value should not be assessed independently from TNM stage, histological subtype, performance status, treatment response, systemic inflammation, and standard serum biomarkers such as CEA or CYFRA 21-1 [18,21,22]. In this setting, elevated or persistently increased PRL could hypothetically indicate a subgroup of patients with more pronounced tumor-related endocrine and inflammatory dysregulation, especially if it coexists with radiological progression, poor clinical status, or increasing conventional biomarkers.

The most clinically realistic approach would be to include PRL in multivariable prognostic models rather than to define a single universal cut-off value. This is in line with current trends in NSCLC in which blood-based risk models are increasingly incorporating tumour markers, inflammatory indices and nutritional parameters to improve prognostic accuracy [22,98]. Experimental data showing that PRL may support cell proliferation and survival through the GHR/JAK2/STAT3 pathway provide a biological rationale for its possible association with aggressive tumor behavior [50]. However, before PRL can be used for risk stratification, future studies must confirm whether it provides independent prognostic information beyond standard clinical, molecular, inflammatory, and serum tumor markers.

### 6.2. Longitudinal Monitoring

The greatest potential clinical value of PRL in NSCLC may lie in longitudinal monitoring rather than in a single baseline measurement. Serial PRL assessment could theoretically help capture changes in disease activity during and after systemic therapy, especially when interpreted together with imaging, symptoms, performance status, and established serum markers such as CEA, CYFRA 21-1, NSE, or SCC antigen [21,99,100]. In this context, the direction of change may be more informative than an isolated value. Persistent elevation, lack of normalization, or secondary increase during follow-up could suggest disease progression, treatment-related endocrine disturbance, systemic stress, or a combination of these mechanisms.

This idea is supported by studies showing that dynamic changes in serum tumor markers may correlate with treatment response and survival outcomes in advanced NSCLC treated with immunotherapy [99,100]. In addition, preliminary observations in metastatic NSCLC patients treated with nivolumab reported hyperprolactinemia during therapy and suggested its association with disease progression [76]. PRL dynamics might be interesting to investigate as an additional monitoring parameter, particularly in patients receiving systemic treatment. However, its use would require standardized sampling time, repeated measurements at predefined clinical points, control of pharmacological confounders, and validation against radiological response, progression-free survival, and overall survival.

### 6.3. Patient Subgrouping

The clinical value of PRL in NSCLC is unlikely to be uniform across all patients. Its interpretation may differ according to sex, age, menopausal status, histological subtype, disease stage, systemic inflammation, and type of anticancer therapy. These variables are important because PRL is strongly influenced by endocrine and inflammatory regulation, while NSCLC itself represents a biologically heterogeneous disease [18,22]. Therefore, future studies should assess PRL separately in clinically defined subgroups rather than only in unselected NSCLC populations.

Particular attention should be paid to patients receiving systemic therapy, especially immunotherapy or chemotherapy, because treatment may modify endocrine and immune responses. Preliminary data in metastatic NSCLC treated with nivolumab suggest that hyperprolactinemia may occur during therapy and may be associated with disease progression [76]. Another potentially relevant subgroup includes patients with advanced disease, cachexia, high inflammatory burden, or poor performance status, in whom elevated PRL may reflect both tumor biology and systemic deterioration. In contrast, patients using opioids, dopamine antagonists, antipsychotics, or metoclopramide should be analyzed separately or excluded from biomarker validation studies because these drugs may independently increase PRL levels.

### 6.4. Comparison with Established Biomarkers

Compared with established serum biomarkers such as CEA, CYFRA 21-1, SCC antigen, and NSE, PRL has several practical advantages, including low cost, easy availability, and the possibility of repeated measurement. However, its main weakness is limited cancer specificity. Classical serum markers are more directly associated with tumor burden or histological subtype, whereas PRL may reflect a broader mixture of tumor-related signaling, systemic inflammation, stress response, endocrine dysregulation, and treatment effects [13,21,99,100].

Therefore, PRL should not be presented as an alternative to CEA, CYFRA 21-1, NSE, SCC antigen, molecular testing, or PD-L1 assessment. Its potential role is more realistic as an additional biomarker used in combination with existing tools. This approach is supported by studies showing that multimarker serum panels, including PRL together with markers such as CEA or CYFRA 21-1, may perform better than single-marker assessment in lung cancer-related analyses [101]. In personalized medicine, the most promising application of PRL would therefore be its integration into broader prognostic or monitoring models, where it could provide complementary information on endocrine-inflammatory tumor-host interactions rather than direct tumor specificity.

**Figure 3:**
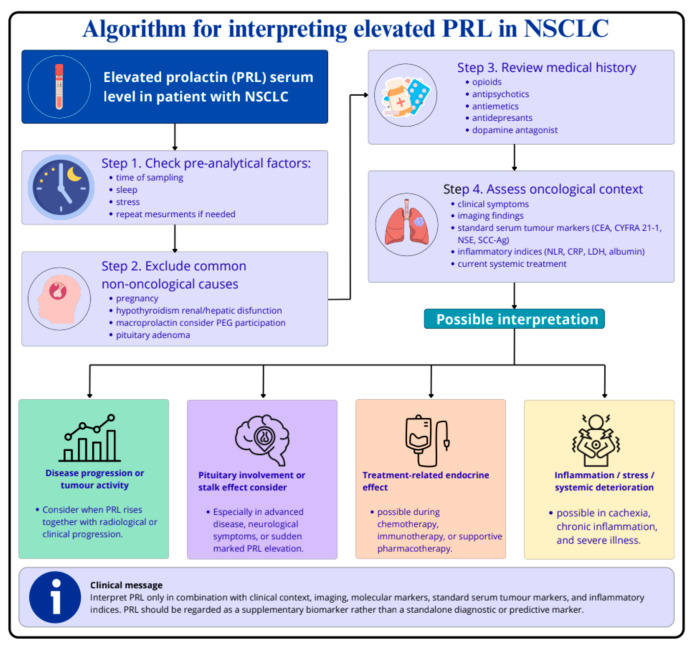
Suggested framework for interpreting prolactin levels in NSCLC.

Overall, the potential clinical utility of PRL in personalized NSCLC management appears to be most realistic when it is treated as a supplementary biomarker rather than an independent clinical tool. Its main value may lie in supporting risk stratification, longitudinal monitoring, and subgroup-specific interpretation, especially when combined with established clinical, molecular, serum, and inflammatory parameters. However, due to its limited specificity and susceptibility to multiple biological, pharmacological, and analytical confounders, PRL requires prospective validation before it can be incorporated into routine clinical decision-making (Figure 3).

## 7. Confounders, Analytical Limitations, and Barriers to Clinical Translation of Prolactin in NSCLC

The interpretation of prolactin as a prognostic biomarker in NSCLC faces significant challenges due to multiple confounding factors and methodological issues, as demonstrated by both clinical and experimental studies [102,103]. Although available data suggest a role for prolactin in NSCLC biology, its clinical significance remains ambiguous [50,76].

### 7.1. Biological Confounders

The physiological variability of prolactin levels, including the impact of stress, circadian rhythm, and sleep disturbances, as well as the lack of standardization in sample collection, has been extensively discussed in previous sections. These factors collectively limit the reliability of PRL as a prognostic biomarker in NSCLC and must be carefully considered in both research and clinical practice (see, Section 4.3.1 and Section 4.3.2) [103,104,105,106].

Metabolic factors and the general condition of the patient are also relevant. Cancer cachexia and metabolic disorders affect the regulation of the hypothalamic–pituitary axis, as observed in advanced cancer stages [106]. Importantly, experimental studies have shown that NSCLC cells can produce prolactin, further complicating the interpretation of its systemic levels as a biomarker [50]. Both in vitro and in vivo models have confirmed the presence of prolactin within tumor tissue and its involvement in regulating cancer cell proliferation [50].

### 7.2. Clinical Confounders

In the NSCLC patient population, comorbidities can significantly influence prolactin levels and lead to erroneous prognostic conclusions [103]. Hypothyroidism is one of the most common causes of secondary hyperprolactinemia via stimulation of thyrotropin-releasing hormone [102]. Renal and hepatic insufficiency, whether as comorbidities or as complications of cancer therapy, result in decreased prolactin clearance and its accumulation [103,107].

Chronic inflammation, characteristic of NSCLC, may affect prolactin secretion through pro-inflammatory cytokines, consistent with its role as an immunomodulatory factor [105]. Notably, prolactin may be directly associated with cancer progression—elevated levels have been correlated with poorer prognosis in lung cancer patients [108]. Rare cases of ectopic prolactin production by lung tumors have also been reported, further complicating differential diagnosis [108].

### 7.3. Pharmacological Confounders

Pharmacotherapy in NSCLC patients is a major source of prolactin disturbances [107]. Metoclopramide and other antiemetics, routinely used during chemotherapy, induce hyperprolactinemia by blocking dopamine receptors [107]. Antipsychotic and antidepressant medications, frequently prescribed in this patient group, also affect the dopaminergic system and may increase prolactin levels [109]. Opioids, commonly administered for cancer pain, also modulate prolactin secretion via the hypothalamic axis [104].

Additionally, as highlighted in previous sections, immunotherapy (e.g., nivolumab) has been associated with a high incidence of hyperprolactinemia, which may correlate with disease progression [76]. This indicates that changes in prolactin levels may result from both tumor biology and anticancer treatment effects.

### 7.4. Analytical and Pre-Analytical Limitations

Methodological limitations are a persistent obstacle in evaluating prolactin as a biomarker in NSCLC [110]. Variability in assay techniques, lack of standardized oncology-specific cut-off values, and the infrequent exclusion of macroprolactin all contribute to inconsistent results and hinder direct comparison between studies [102,103]. While prolactin levels tend to be higher in NSCLC patients than in healthy controls, its clinical utility emerges primarily when interpreted alongside established markers such as CEA or CYFRA21, rather than as an isolated parameter [4]. The timing of sample collection and storage conditions remains critical for assay reliability [102,105].

An important analytical limitation identified in the current literature concerns the assessment of prolactin receptor isoforms. Experimental evidence indicates that the long PRLR isoform is the principal mediator of prolactin-driven oncogenic signaling, promoting tumor progression, metastatic dissemination, and resistance to therapy. However, most routinely used clinical immunoassays, including enzyme-linked immunosorbent assays (ELISA), chemiluminescent immunoassays, and standard immunohistochemical protocols, are unable to differentiate between the long and short PRLR variants. This limitation arises because the antibodies employed in these assays typically recognize epitopes located within receptor regions shared by multiple isoforms, resulting in the measurement of total PRLR expression rather than isoform-specific expression patterns. As a consequence, biologically significant differences in receptor signaling may remain undetected, potentially limiting prognostic accuracy and patient stratification.

Bridging this bench-to-bedside gap will require the development and clinical validation of isoform-specific diagnostic methodologies. Potential approaches include monoclonal antibodies directed against unique domains of the long PRLR isoform, isoform-specific immunohistochemical panels, and transcript-based molecular techniques such as quantitative RT-PCR, digital PCR, and RNA sequencing capable of distinguishing alternatively spliced PRLR transcripts. The implementation of these technologies could improve prognostic assessment, facilitate more precise risk stratification, and support the development of personalized therapeutic strategies targeting prolactin-dependent signaling pathways.

### 7.5. Barriers to Clinical Implementation

Despite the growing body of experimental and clinical data, the use of prolactin as a prognostic biomarker in NSCLC remains limited [50]. As previously discussed, molecular studies indicate that prolactin can promote NSCLC cell proliferation through activation of the GHR/JAK2/STAT3/VEGF pathway, supporting its potential involvement in disease progression [50]. Furthermore, genetic (Mendelian randomization) studies have shown that higher prolactin levels are associated with an increased risk of lung cancer, further supporting its potential role in carcinogenesis [68].

However, it should be emphasized that available clinical studies are few and involve small patient cohorts, limiting their statistical power and generalizability [103]. There is also a lack of external validation and unequivocal evidence that prolactin measurement influences therapeutic decisions or improves patient outcomes [4]. Ultimately, the greatest challenge remains the integration of biological data into clinical practice, as even statistically significant associations do not always translate into clinical utility [111].

## 8. Conclusions

Prolactin is not yet an established biomarker in NSCLC, but current evidence suggests that it may have potential as a supplementary circulating marker, particularly for prognosis and post-treatment monitoring. Its biological relevance is supported by its involvement in pathways related to tumour proliferation, survival, invasion, angiogenesis, immune modulation and possible therapy resistance.

In clinical practice, PRL should not be interpreted as a standalone diagnostic or predictive marker. Its levels may be influenced by many confounders, including stress, circadian rhythm, inflammation, endocrine disorders, cachexia, pituitary metastases, macroprolactin and medications such as opioids, antipsychotics or antiemetics. Therefore, PRL should be assessed only in combination with clinical data, imaging, molecular markers, standard serum tumour markers and inflammatory indices.

The most realistic future role of PRL in NSCLC lies in multimarker prognostic models and longitudinal monitoring after systemic treatment, including chemotherapy. Serial changes in PRL may potentially help identify patients at higher risk of progression, but this requires confirmation in large prospective studies with standardized sampling, assay methods and adjustment for confounding factors.

In conclusion, prolactin is a promising but still unvalidated candidate biomarker in NSCLC. Its clinical use should remain investigational until robust evidence confirms its independent prognostic value and practical utility in personalised patient management.

## Figures and Tables

**Table 1:** Summary of clinical evidence, cohort characteristics, and prolactin level dynamics in lung cancer populations.

Study Type	Sample Size	Demographics	Clinical Stage & Histology	Treatment Status	Observations	Ref.
Autopsy Cohorts	In total, 1% to 4% of advanced terminal cancer cases.	Elderly patients (predominantly 60 to 70 years old).	Advanced metastatic disease with direct pituitary involvement; accounts for ~1% of all pituitary tumors.	Post mortem analysis (end-stage disease).	Lung and breast cancers combined account for approximately 50% of all pituitary metastases. Associated with an increasing frequency of anterior hypopituitarism.	[63]
Clinical Cohorts	In total, ~1% clinically diagnosed during lifetime.	Mean age of ~49.5 years; strong male predominance (64% of cases).	Advanced NSCLC (Adenocarcinoma, Squamous Cell Carcinoma); Stage IVB (M1 category).	Active disease, pre-intervention phase, or progression during targeted therapies (e.g., crizotinib resistance).	Markedly elevated PRL (often >110–200 ng/mL) driven by the “stalk effect” (mechanical compression blocking dopamine) or direct tumor tissue invasion. Correlates with secondary mutations (e.g., ALK-I1171S).	[85,89]
Ectopic Neuroendocrine Cohorts	Individual advanced cases (highly metabolic thoracic masses).	Adult patients presenting with exceptionally high tumor burden.	Advanced Pulmonary Neuroendocrine Tumors (NETs); presenting as large thoracic masses (e.g., 75 × 87 × 106 mm).	Pre-operative diagnostic phase vs. Post-radical surgical resection.	Extreme “tumoral range” hyperprolactinemia (330–832 ng/mL) caused by ectopic GHRH hypersecretion (38,088 ng/L) driving secondary lactotroph hyperplasia. Post-op: Rapid drop to 1.9 ng/mL within 5 days, stabilizing at 3.6 ng/mL in the long term.	[90]
Immunotherapy Cohorts	Target populations monitored on immune checkpoint inhibitors.	Adult patients receiving anti-PD-1/PD-L1 checkpoint blockades.	Advanced NSCLC showing partial response (PR) or stable disease.	On-treatment monitoring and post-treatment follow-up (up to 17 months after initial dose).	Stable/Normal PRL levels. The stability of PRL helps differentiate immune-related adverse events (e.g., Isolated ACTH Deficiency/hypophysitis) from mechanical pituitary metastases.	[91]

ACTH, adrenocorticotropic hormone; ALK, anaplastic lymphoma kinase; GHRH, growth hormone-releasing hormone; M1, distant metastasis; NET, neuroendocrine tumor; NSCLC, non-small cell lung cancer; PD-1, programmed cell death protein 1; Post-op, post-operative; Pre-op, pre-operative; PRL, prolactin.

**Table 3:** Predictive value of hormonal parameters in monitoring NSCLC treatment response [50,64,67,76].

Parameter/Molecule	Predictive Value and Treatment Monitoring	Impact on Therapeutic Response and Mechanism
Prolactin ↑	Early indicator of poor response to nivolumab immunotherapy.	Induces chemotherapy resistance (cisplatin, doxorubicin) via activation of DNA repair systems (ATM) and interaction with heat shock proteins (HSP90).
Leptin ↑	Favorable prognostic factor, particularly in women with NSCLC.	Correlates with improved OS following the administration of PD-1/PD-L1 checkpoint inhibitors.
Activin A ↑	Marker of resistance to selected forms of immunotherapy.	Associated with a lack of durable clinical benefit following atezolizumab treatment and the risk of developing cancer-related sarcopenia.
FSH/LH ↑	Indicator of increased tumor invasiveness, particularly significant in patients over 50 years of age.	Stimulates tumor aggressiveness and metastatic potential by inhibiting the expression of HO-1.
ACTH ↓ (with normal prolactine)	Signal of isolated ACTH deficiency (IAD) occurrence as an immune-related adverse event.	Often correlates with good disease control and achievement of PR to PD-1 inhibitor therapy.

Abbreviations: ↑—Increase; ↓—Decrease; PR—Partial Remission; OS—Overall Survival; ATM—Ataxia-Telangiectasia Mutated Kinase; HSP90—Heat Shock Protein 90; IAD—Isolated ACTH Deficiency; HO-1—Heme Oxygenase-1.

## Data Availability

No new data were created or analyzed in this study. Data sharing is not applicable to this article.

## Abbreviations

The following abbreviations are used in this manuscript:

ACTH	adrenocorticotropic hormone
ADH	antidiuretic hormone
ALK	anaplastic lymphoma kinase
ATM	ataxia-telangiectasia mutated kinase
CEA	carcinoembryonic antigen
CRP	C-reactive protein
CYFRA 21-1	cytokeratin 19 fragment antigen 21-1
DA	dopamine
DFS	disease-free survival
EGFR	epidermal growth factor receptor
EMT	epithelial–mesenchymal transition
ERBB2	erb-b2 receptor tyrosine kinase 2
ERK	extracellular signal-regulated kinase
FSH	follicle-stimulating hormone
GH	growth hormone
GHR	growth hormone receptor
GHRH	growth hormone-releasing hormone
GnRH	gonadotropin-releasing hormone
HER2	human epidermal growth factor receptor 2
HO-1	heme oxygenase-1
HPA axis	hypothalamic–pituitary–adrenal axis
HSP90	heat shock protein 90
IAD	isolated ACTH deficiency
ICI	immune checkpoint inhibitor
IHC	immunohistochemistry
IL-6	interleukin-6
JAK	Janus kinase
JAK2	Janus kinase 2
JAK/STAT	Janus kinase/signal transducer and activator of transcription
Ki-67	marker of cellular proliferation
KRAS	Kirsten rat sarcoma viral oncogene homolog
LDH	lactate dehydrogenase
LH	luteinizing hormone
MAPK	mitogen-activated protein kinase
MAPK/ERK	mitogen-activated protein kinase/extracellular signal-regulated kinase
MET	mesenchymal–epithelial transition factor
MMP	matrix metalloproteinase
MMP-2	matrix metalloproteinase-2
MMP-9	matrix metalloproteinase-9
mTOR	mechanistic target of rapamycin
N	regional lymph node status in TNM classification
NET	neuroendocrine tumor
NGS	next-generation sequencing
NLR	neutrophil-to-lymphocyte ratio
NSE	neuron-specific enolase
NSCLC	non-small cell lung cancer
NTRK	neurotrophic tyrosine receptor kinase
OS	overall survival
PD-1	programmed cell death protein 1
PD-L1	programmed death-ligand 1
PEG	polyethylene glycol
PFS	progression-free survival
PI3K	phosphoinositide 3-kinase
PI3K/AKT	phosphoinositide 3-kinase/protein kinase B
PR	partial response/partial remission
PRL	prolactin
PRLR	prolactin receptor
RET	rearranged during transfection proto-oncogene
ROS1	ROS proto-oncogene 1, receptor tyrosine kinase
SCC-Ag	squamous cell carcinoma antigen
SHH	Sonic Hedgehog
STAT	signal transducer and activator of transcription
STAT3	signal transducer and activator of transcription 3
STAT5	signal transducer and activator of transcription 5
STAT5A	signal transducer and activator of transcription 5A
STAT5B	signal transducer and activator of transcription 5B
T	primary tumor status in TNM classification
TIDA neurons	tuberoinfundibular dopamine neurons
TME	tumor microenvironment
TNF-α	tumor necrosis factor alpha
TNM	tumor, node, metastasis classification
TRH	thyrotropin-releasing hormone
Treg	regulatory T cells
VEGF	vascular endothelial growth factor
